# The Mechanisms of Traditional Chinese Medicine Underlying the Prevention and Treatment of Parkinson's Disease

**DOI:** 10.3389/fphar.2017.00634

**Published:** 2017-09-19

**Authors:** Xiaoliang Li, YaNan Zhang, Yu Wang, Jing Xu, Ping Xin, YongHai Meng, Qiuhong Wang, Haixue Kuang

**Affiliations:** ^1^Key Laboratory of Chinese Materia Medica (Ministry of Education), Heilongjiang University of Chinese Medicine Harbin, China; ^2^Science of Chinese Materia Medica, Jiamusi College, Heilongjiang University of Chinese Medicine Jiamusi, China; ^3^Science of Processing Chinese Materia Medica, College of Pharmacy, Guangdong Pharmaceutical University Guangzhou, China

**Keywords:** Parkinson's disease, traditional Chinese medicine, oxidative stress, neuronal apoptosis, mitochondrial dysfunction

## Abstract

Parkinson's disease (PD), characterized with bradykinesia, static tremor, rigidity and disturbances in balance, is the second most common neurodegenerative disorder. Along with the largely aging population in the world, the incidence is increasing year by year, which imposes the negative impacts on patients, their families and the whole society. Traditional Chinese medicine (TCM) has a positive prospect for the prevention and cure of PD due to its advantages of less side effects and multi-target effects. At present, the pathogenesis of PD is not yet fully discovered. This paper elaborates the mechanisms of TCM underlying the prevention and treatment of PD with regards to the inhibition of oxidative stress, the regulation of mitochondrial dysfunction, the reduction of toxic excitatory amino acids (EAA), the inhibition of neuroinflammation, the inhibition of neuronal apoptosis, and the inhibition of abnormal protein aggregation.

## Introducion

Parkinson's disease (PD), the second most common neurodegenerative disorder among the aging population after Alzheimer disease, is characterized by a combination of typical motor symptoms that include akinesia, rigidity, bradykinesia, and often resting tremor (Michel et al., 2016; Rizek et al., 2016). The pathological changes in several areas of the brain are mainly marked by the degeneration of dopaminergic neurons (Damier et al., 1999). The disease is recognized as one of the most common, difficult and complicated neurological disorders identified by WHO. With the global trends in aging, the incidence of PD has increased year by year and the prevalence rate is high up to 1–2% among the elderly over the age of 65 years (Alves et al., 2008; Wang et al., 2012).

Clinically, the PD patients are usually treated with levodopa, dopamine receptor agonist, monoamine oxidase B inhibitors and other types of drugs. The clinical symptoms of the disease are mitigated by supplementing dopamine or reducing the degradation of it. However, the pathogenesis of PD is still not very clear, so that the efficacy of these drugs is not ideal and the unpleasant side effects are apparent after long-term administration such as the motor complications (Stocchi and Marconi, 2010), nausea, constipation, headache, and sleep disorder, etc. (Borovac, 2016) which would negatively influence the quality of life of the patients (Kum et al., 2011; Qin and Wu, 2016).

Considering the long-term side effects of western medicines, many patients are searching for a more safe and effective alternative treatment for PD. TCM has been used for centuries to treat diseases such as the tremor of head and hands, which is similar to the modern PD. The therapy of TCM for tremor, either single herb or herbal formula, could be traced back to the Huangdi Neijing (Huangdi's Internal Classic), the earliest existing classics in Chinese medicine (Zhang Y. et al., 2014). Up to the present, TCM is still very popular in the treatment of PD in some Asian countries such as China, Korea, and Japan (Kum et al., 2011). And much more attentions have been drawn to the TCM active ingredients showing a definite effect and a clear structure.

**Figure 1:**
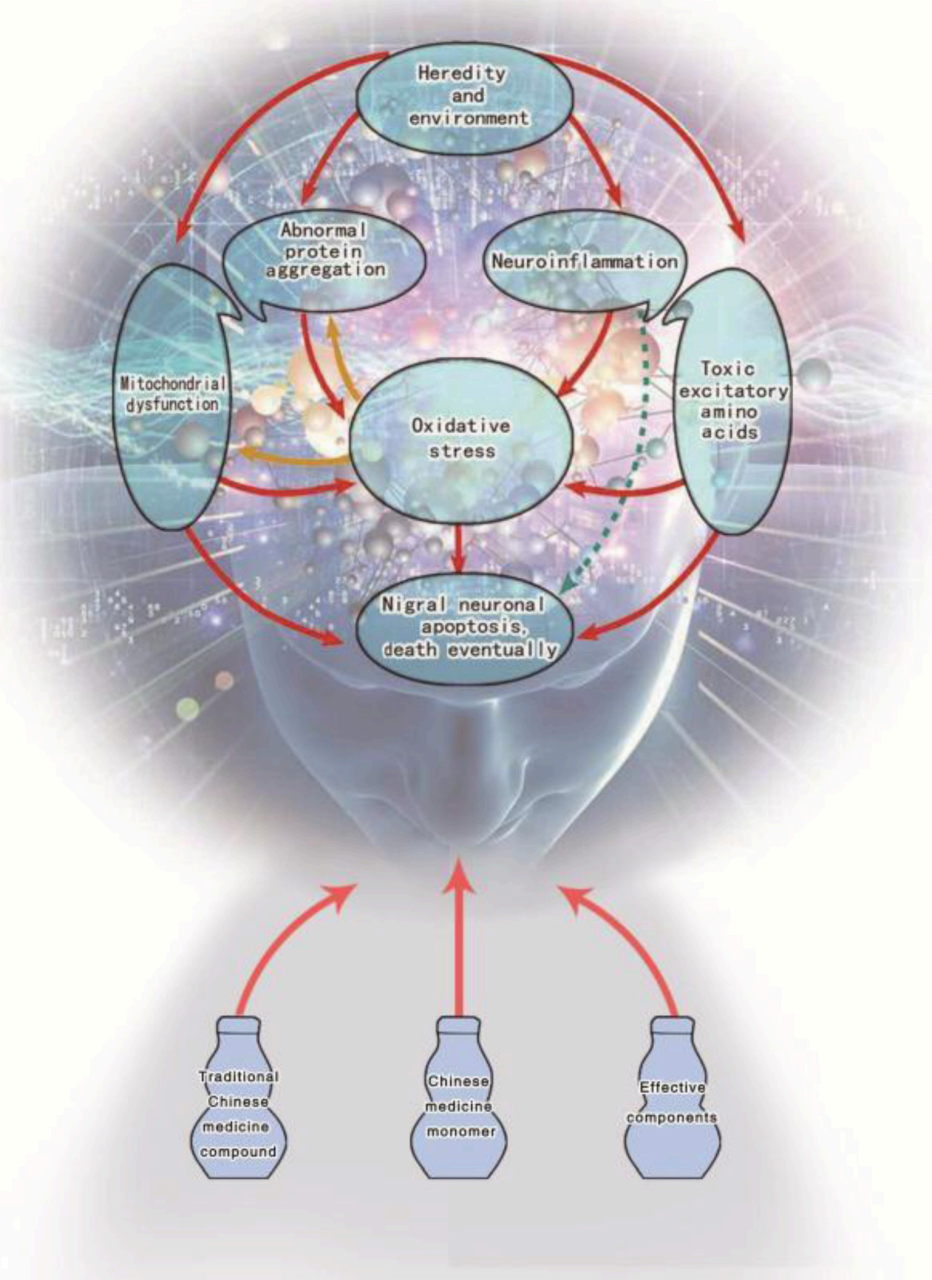
Potential mechanisms of traditional Chinese medicine for Parkinson's disease therapy.

So far, there is still no exact cure for PD due to its diversity of etiology and complexity of symptoms. So, it is still a hot spot in the research of PD to figure out effective treatment methods and drugs. In Figure 1, it is clearly stated that under the effects of genetic or environmental factors the activation of any links (mechanism) can lead to injury or even death of dopaminergic neurons in the substantia nigra. Therefore, taking pathological mechanism of PD as the main stream, this paper attempts to summarize and analyze the mechanism of TCM which includes Chinese herbal compounds, herbal medicines, herbal formulations and TCM active ingredients in terms of prevention and treatment of PD.

## Central nervous system activity

### The inhibition of oxidative stress

The etiology and pathogenesis of PD so far have not been completely elucidated, but it has been generally acknowledged that the improvement of oxidative stress is one of the most important pathophysiological mechanisms (Avila et al., 2009; Chen et al., 2009; Sanyal et al., 2009).

Oxidative stress is incurred by the increase of free radicals in the organism, while the eliminative ability of free radicals decreased at the same time. There will be excessive free radicals in the body, which will be damaging cell components eventually (Sompol et al., 2008). PD patients are in a state of oxidative stress. In the substantia nigra of PD patients, the elevated concentration of ferric ion, weaken mitochondrial function and anomaly protection system of antioxidant (Such as low molecular free radical scavenger glutathione (GSH) and free radical scavenging enzyme SOD, GSH-Px) have contributed to the acceleration of oxidative stress and excessive generation of oxygen free radicals. Thereby, large amount of lipid peroxide, such as Malondialdehyde (MDA), hydroxyl, carbonyl, etc., will cause cell death, which leads to neuronal apoptosis ultimately (Wu et al., 2009; Wang et al., 2012).

Mainly distributed in grape, ginkgo, rhubarb, hawthorn, hypericum erectum, and other plants, procyanidin is a kind of bioflavonoid with special molecular structure, and it is currently recognized as the most effective natural antioxidant in the human body. After entering the body, procyanidin can be absorbed rapidly and directly involved in the physiological functions of the body, which shows stronger ability to scavenge hydroxyl radical, superoxide anion radical and other active oxygen. The ability of 100 mg/L procyanidin to scavenge superoxide anion and hydroxyl radical was 6.51 and 4.26 times higher than vitamin C, respectively (Bagchi et al., 1997). Procyanidin at dose of 400 mg/kg could significantly improve the grip function of PD mice which was established by intraperitoneal injection of 1-methyl-4-henyl-1,2,3, 6-tetrahydropyridine (MPTP). Meanwhile, the surging of MDA content, the decrease of SOD and GSH-Px activity in the substantia nigra of PD mice was effectively inhibited (Liang and Zhang, 2016). In the study of *Clerodendranthus spicatus*, the researchers found that the total flavonoids were the major active constituent which elicited affirmative effects on antioxidant activity and cleaning free radical. The total flavonoids of *Clerodendranthus spicatus* produced a markedly protective effect on the PD rat model and cell model induced by 6-Hydroxydopamine (6-OHDA). The protective effects may be related to reducing cell damages through reducing the level of oxidative stress (You et al., 2015).

In summary, oxidative stress plays a crucial role in the occurrence and deterioration of PD, and we can achieve the purpose of prevention and treatment against it by resisting oxidative stress. Many TCM or its effective components may act as a potential antioxidant. Therefore, the idea of screening TCM to delay the progression of PD has attracted the attention from many researchers. An overview of the improvement of oxidative stress is described in detail below, and the additional information is shown in Table 1.

**Table 1:** Effect of TCM on Oxidative Stress Responses in the Model of PD.

**TCM**	**Oxidative stress indexes**					**Model**	**References**
	**SOD**	**MDA**	**GSH-PX**	**GSH**	**CAT**		
Lycopene	↑	↓	↑		↑	Mice	Liu et al., 2013
Protocatechuic acid	↑	↓				Rat	Zhang Q. et al., 2015
Proantho cyanidins	↑	↓	↑			Mice	Liang and Zhang, 2016
Protocatechuic acid and chrysin	↑	↓			↑	PC12 cells	Zhang Z. J. et al., 2015
Green tea polyphenols	↑	↓				Mice	Chen et al., 2013
Total flavonoids of *Clerodendranthus spicatus*	↑	↓		↑	↑	Rat	You et al., 2015
Pine bark extract	↑	↓		↑		Mice	Lu et al., 2014
Petroleum ether extract of *Ficus religiosa* (L.) leaves	↑	↓		↑	↑	Mice	Bhangale and Acharya, 2016
Powder *of Gastrodia elata*	↑	↓	↑	↑		Rat	Chen et al., 2014
*Ganoderma lucidium* spore	↑	↓	↑	↑		Rat	Bao, 2014
Zhenganxifeng decoction		↓				Rat	Li X. M. et al., 2016
Baichanting compound	↑	↓	↑			Mice	Ren et al., 2015
Gui ling Pa An Wan	↑	↓		↑		Rat	Meng et al., 2014a

### The regulation of mitochondrial dysfunction

Abnormal morphology and dysfunction of mitochondrial are one of the important pathological mechanisms of PD.

Mitochondria, as the “power plant” and “energy conversion station” of cells, regulates the process of gene expression and apoptosis. Recent reports have suggested that mitochondrial dysfunction is closely related to a variety of neurodegenerative diseases including PD (Exner et al., 2012; Liu et al., 2012; Feng and Wu, 2014).

The decrease of mitochondrial complex I activity of substantia nigra neurons in autopsy of PD was firstly found by Shoffner et al. (1991). Shortly afterwards, Parker et al. found that the platelet mitochondrial complex I activity in patients with PD was also reduced (Parker et al., 1989). After the inhibition of mitochondrial complex I, there are obvious obstacles to the production of energy, which lead to a series of secondary reactions, even cell death occurs. Insufficient synthesis of ATP can also cause protein and lipid degradation. The degraded products may trigger or produce oxidative metabolic reactions, which aggravate the damage of substantia nigra. The production of large amount of reactive oxygen species elicited by the inhibition of complex I can drive its activity to continue to decline, thus form a vicious circle (Tada-Oikawa et al., 2003; Xiong et al., 2012).

MPTP which is an inhibitor of mitochondrial respiratory chain complex I can selectively damage dopaminergic neurons in the substantia nigra pars compacta. The mice are injected with MPTP to produce mitochondrial dysfunction and oxidative stress, which create similar clinical symptoms and pathological changes in PD (Blesa and Przedborski, 2014). In a study, locomotor activity, pole and rotarod test were used to evaluate the effects of Qianzheng San extract to the dyskinesia induced by MPTP. Compared with the model group, the MPTP-treated mice laid out a significant reduction in locomotor activity and ultrastructure of substantia nigra neuron was obviously harmed. However, Qianzheng San extract treatment largely increased autonomic activities, prolonged incubation period and shortened the pole-climbing time (*P* < 0.05), and reduced the impairment of ultrastructure of substantia nigra neurons. On the other hand, electron microscopy showed that the ultrastructure of substantia nigra neurons was ameliorated effectively and the high degree of mitochondrial damage was alleviated remarkedly by treatment of Qianzheng San extract. All these experimental results reveal that Qianzheng San extract may play a neuroprotective role through improving mitochondrial functions (Li et al., 2015). Catalpol, which is relatively abundant in the TCM such as Radix Rehmanniae and Radix Scrophulariae, is a small molecule compound of iridoids. It also showed that it has protective effects on mice brain mitochondrial damage induced by rotenone, partly through enhancing the activities of complex I, increasing the content of GSH, lowering the loss of mitochondrial membrane potential and restraining the release of LDH (Shi et al., 2012).

Baicalein, a well-known flavonoid compound isolated from dried roots of Scutellaria baicalensis, has been applied extensively as an antioxidant and anti-inflammatory agent (Ge et al., 2017). In recent years, with continuous studies on its mechanisms, it has been found that baicalein has some effect on the improvement of clinical symptoms and neuroprotection in neurodegenerative diseases such as PD. The protective effect of baicalein on mitochondria may be one of the pharmacological targets of neuroprotection against PD. The study confirmed that exposure of PC12 cells to 0.15 mM H_2_O_2_ for 20 min resulted in mitochondrial damage and cell apoptosis. And pre-treatment of PC12 cells with different concentrations of baicalein greatly cut down the cell viability loss. The protective effect of baicalein on mitochondrial function was related to inhibition of ROS production and the regulation of Bcl-2 family members first, and these regulations might adjust the mitochondrial membrane permeability, attenuate cytochrome c release to cytosol (Zhang et al., 2010).

### The reduction of toxic EAA

In pathological conditions, glutamate (Glu) can produce the effects of excitotoxicity on nerve cells. The relationship between neurotoxicity of Glu and pathogenesis of PD has received elevating attention. At present, the treatment of PD with Glu release inhibitor has become one of the hottest spots in the research.

In recent years, a growing number of evidences suggests that in addition to dopamine (DA) and acetylcholine (Ach), there are also many other neurotransmitters such as Glu, gamma-aminobutyric acid (GABA) and enkephalin etc., and they can interact with each other in the substantia nigra and striatum (Papa and Chase, 1996). Under normal conditions, Glu creates excitatory effect on nerve cells, but it demonstrates toxic effects when DA neurons are fully or partially degenerated (Vital et al., 2003). The concentration of Glu in normal brain cells is close to 10 μmol/L, while the extracellular concentration is about 0.6 μmol/L. When the extracellular Glu concentration reaches 25 μmol/L, it can damage the cerebral cortex and hippocampus (Caragine et al., 1998; Zhou et al., 2003). The toxicity of EAA (Glu, aspartate) is mainly reflected in the activation of the corresponding receptor (NMDA-R, AMPA-R, KA-R) which mediate acute osmotic swelling or delayed injury of nerve cells. It was found that local and systemic application of EAA receptor antagonists could lower or prevent motor symptoms of the PD model rats induced by 6-OHDA, and postpone its the process of neurodegenerative (Hallett and Standaert, 2004). It was separately reported that low dose of NMDA receptor antagonist MK-801 combined with levodopa could enhance the efficacy and prolong its duration of action. Clinically, motor fluctuations and dyskinesia, caused by long term use of levodopa, can be effectively treated by MK-801. Anticholinergics are one of NMDA receptor non-competitive antagonists. Amantadine, a drug that has been used for years to moderately intervene symptoms of PD, has also been shown to be an NMDA receptor antagonist (Strugstad and Sager, 1998; Blesa and Przedborski, 2014).

Tetrandrine (Tet), a class of bisbenzylisoquinoline extracted from the roots of Radix stephania tetrandrae, (Wong et al., 2017) is a new type of reversible inhibitor of P-glycoprotein. The level of L-dopa in the brain can be increased by reversible P-glycoprotein inhibitors, which is conductive to clinical efficacy of neurodegenerative diseases, including PD (Schinkel, 1999). The researchers used Tet combined with GSH and L-dopa to explore the therapeutic mechanism of PD rats induced by 6-OHDA. By detecting the aspartate (Asp) and Glu in the affected side of striatum, it was evident that compared with the model group, the concentration of Asp was dramatically downgraded in the GSH treatment group; The level of Glu in the GSH + Tet treatment group was much lower than that in the GSH treatment group; The concentration of Glu and Asp in the L-dopa treatment group was notably higher than that in the model group; The concentration of Glu and Asp in the GSH + L-dopa + Tet treatment group was considerably lower than the model group. The above results show that Tet, by the means of increasing the concentration of anti-PD drugs in the brain, can protect the brain neuron from the toxic effect of EAA (Jin and Bao, 2010). In a separate study, Glu treatment largely increased LDH release and produced a great deal of NO in primary cultured rat brain neurons. While Baicalein, at 3.5 μmol·L^−1^, could exert neuroprotective effects against Glu stimulation by reducing the generation of LDH and NO (Yu et al., 2012). Puerarin, a kind of flavonoid compound, was extracted from Puerariae Radix. It stated neuroprotective effects on a variety of brain damage by sharply reducing the content of EAA. The results demonstrated that puerarin can promote the expression of Glu decarboxylase mRNA in rats with cerebral ischemia and increase the contents of cerebral GABA to antagonize the toxic effect of EAA (Huang and Wang, 2015).

### The inhibition of neuroinflammation

Neuroinflammation is a common and important pathological mechanism in nervous system diseases and different neurological diseases are involved in neuroinflammation at some stage. At present, it is believed that neuroinflammation was involved in an important cascade reaction in neuronal degeneration of PD (Niranjan, 2014).

Along with aging, dysregulation of immune and inflammatory will gradually appear in the body, and the activation of microglia is considered to be related to the pathogenesis of PD nerve degeneration. When the central nervous system suffers from exogenous antigens stimulus, such as pathogenic microorganisms or foreign bodies, microglia will be rapidly activated. Then, the activated microglia cells can secrete various cytokines such as IL-1β, IL-2, IL-4, IL-6, TNF-α, and IFN-γ, etc. (Hunot and Hirsch, 2003).

The increased levels of cytokines can cause inflammatory response and neuronal damage, induce the cell to undergo programmed death by increasing the level of nitric oxide (NO) in the brain and lead to the onset of neurodegenerative diseases eventually (Mosley et al., 2006; Béraud et al., 2013). Among them, IL-1β and TNF-α appear especially important because they can promote macrophages and other cells to secrete IL-6, IL-8 and other cytokines. For the animal model of PD induced by the neurotoxin such as MPTP and 6-OHDA, there is obvious activation of microglia in the early stage of degeneration of dopaminergic neurons (Barnum and Tansey, 2010; Miller et al., 2011). The enhancement of the expression of IL-1β, TNF-α and other inflammatory cytokines are observed in the nigrostriatal system of PD patients at autopsy, mainly in activated microglia, and the expression of inflammatory factors is positively correlated with the loss of DA neurons (Miklossy et al., 2006; Tansey and Goldberg, 2010). In addition, up-regulation of cyclooxygenase-2 (COX-2) expression is also vital to immunity and inflammatory responses immunity and inflammatory responses. As an inflammatory response gene, it is involved in the inflammatory response of the body, and generated neuronal apoptosis in the pathological process of PD (Wei et al., 2009; Yu et al., 2012).

In recent years, some progress has been made in the research of anti-inflammatory TCM in the treatment of PD. The protective effect of triptolide on dopaminergic neurons in MPP^+^-induced hemiparkinsonian rats may be concerned to the inhibition of microglial cell activation (Hirsch et al., 2005; Gao et al., 2008). Curcumin can effectively antagonize the loss of dopaminergic neurons in the parkinsonian mouse model caused by MPTP. Its mechanism is associated with the decrease of the active oxygen content of dopaminergic neuron and inhibition of inflammation (Pan et al., 2007). Through the antioxidant and anti-inflammatory effects, celastrol also can efficiently prevent or delay the progression of PD (Faust et al., 2009; Zhang et al., 2012). In the study of Polygona-Polysaccharose on PD, it was found that the expression of Peroxisome proliferator-activated receptor-γ (PPAR-γ) was up-regulated in treatment group as compared with model group. PPAR-γ is a class of ligand-activated type 2 nuclear transcription factor belonging to the nuclear receptor superfamily. It has neuroprotective effects and attenuate the neuronal damages from neurodegenerative diseases such as Alzheimer's disease, PD, cerebral ischemia and multiple sclerosis. Meanwhile, the study results about Polygona-Polysaccharose also revealed that the mechanism might be related to the up-regulation of PPAR-γ expression, thereby inhibiting the inflammatory reaction and promoting the regeneration of dopaminergic neurons (Chen et al., 2010). Polyphenols from toona sinensis seeds (PTSS) can exert the protective effect to DA neurons of substantia nigra of PD rats by reducing the number of microglia and astrocytes in the substantia nigra and down-regulating expression levels of protein and mRNA of inflammatory factors COX-2 and TNF-α (Li X. J. et al., 2016). Parthenolide, as an active ingredient obtained from Chinese herbs tansy, possesses extensive biological functions, such as anti-inflammation, antioxidation et al., and it also has apparent protective effects against the damage of DA neurons induced by MPTP in substantia nigra. The research showed that compared with the mice in control group, the model mice represented the typical symptoms of PD. The numbers of COX-2, PGE2 and iNOS positive cells were reduced noticeably (*P* < 0.01), the number of TH-positive neurons in substantia nigra was decreased from 58 to 27% after the intervention with parthenolide. Taken together, the protective effect of parthenolide for dopaminergic neurons may be related to its activity as an anti-inflammatory in the expression of COX-2, PGE2, and iNOS in substantia nigra of PD mice model (Zhang H. et al., 2015).

### The inhibition of neuronal apoptosis

With the in-depth study, the researchers find that another way of loss of dopaminergic neurons is abnormal apoptosis (Golpich et al., 2015). Apoptosis may be one of the most important factors in the death of dopamine neurons, and accelerate the occurrence and progression of PD (Valdeolivas et al., 2013). Energy consumption of normal activities of brain cells are derived directly from aerobic energy, and there is little energy storage. Once the brain damage occurs, it will cause nerve cell apoptosis or death (Kermer et al., 1999; Li R. et al., 2013). Apoptosis, which is an active programmed cell death, is the terminal phenomenon of gene-induced biological cascade reaction under the stimulation of both *in vitro* and *in vivo*. The Bcl-2 family is a kind of important apoptosis-regulatory genes. It is divided into two categories: anti-apoptosis gene (such as Bcl-2, Bcl-xL, Bcl-w, Bcl-1, etc.) and pro-apoptosis gene (such as Bax, Bak, Bad, Bid, etc.). Bcl-2, located in the outer mitochondrial membrane primarily, can counter pro-apoptotic factor. The early phase of the apoptotic cascade depends mainly on the balance between the pro- and anti-apoptotic proteins of the Bcl-2 family, while the Bcl-2/Bax ratio is regarded as a better predictor of apoptosis than the expression of either Bcl-2 or Bax alone (Sa et al., 2015; Wu et al., 2015). Studies have shown that the overexpression of bcl-2 can break off the apoptosis of various nerve cell, can also restrain the toxic effects of MPTP and 6-OHDA on dopaminergic neurons, thus reducing the apoptosis of dopaminergic neurons in substantia nigra (Burke, 2011; Chen et al., 2015b).

Studies indicated that Guiling pa'an Wan (Chinese patent medicine) could improve pathology and behavior of the PD model rats induced by 6-OHDA. Based on this background, the mechanism of action was further studied. After treatment with Guiling pa'an Wan, the expression of Bcl-2 and Bcl-2/Bax ratio was increased while Bax and Caspase-3 expression were dropped in substantia nigra neurons, which showed that Guiling pa'an could provide protection for dopaminergic neurons by reducing the apoptosis of nerve cells in PD rats (Meng et al., 2014b; Zhang H. Z. et al., 2016). Some flavonoids have good effect on the prevention and treatment of PD. Puerarin can inhibit the expression of p53, Bax, PUMA and caspase 3 via the activation of pi3k/akt signaling pathway in SH-SY5Y cells stimulated by MPP^+^ (Zhu et al., 2012). In 6-OHDA-induced neurotoxicity in a dopaminergic cell line, SN4741 cells, isoliquiritigenin can significantly ameliorate the expression of Bcl-2 and lower expression of Bax and the release of cytochrome C, which can be reversed by the inhibitor of PI3K/Akt/PKB (Hwang and Chun, 2012; Qin and Wu, 2016). Ginkgo biloba Pingchan Recipe has protective impacts on dopaminergic neurons in PD model mice induced by MPTP. The researchers, through SH-SY5Y cell model induced by MPP^+^, made a further study of its mechanism. After the drug treatment of Ginkgo biloba Pingchan Recipe, the proliferation speed of the cells was accelerated while apoptosis was substantially plunged, and the expression levels of apoptosis related gene PARP and PTEN were vastly declined (Wu et al., 2016). As a tumor suppressor gene, PTEN regulates the cell division cycle by preventing fast cell growth and division or uncontrolled cell division (Li and Yang, 2012; Li D. et al., 2013; Zhang G. et al., 2014). Geniposide, an iridoid glycoside compound extracted from the TCM Gardenia jasminoides Ellis fruit, exerts neuroprotective effects by alleviating inflammation responses and oxidative damages (Zhao et al., 2016). The behavioral experiment including rotarod and swimming trials indicated that the Geniposide could substantially improve the abnormal behavior caused by MPTP. Meanwhile, the number of TH positive neuron sharply increased (*P* < 0.001) and the apoptotic neurons fell (*P* < 0.001) after treatment with Geniposide. Thereby suggesting that Geniposide has protective effect on dopaminergic neurons in substantia nigra of PD mice model induced by MPTP, and its mechanism may be related to the inhibition of neuronal apoptosis (Chen et al., 2015a).

### The inhibition of abnormal protein aggregation

Currently, impaired degradation of misfolded and aggregated proteins has been proposed to play a key role in the pathogenesis of PD (Le and Chen, 2009). The abnormal deposition of protein in brain tissue is characteristic of several age-related neurodegenerative diseases, such as PD. Although, the composition and position (i.e., intra- or extracellular) of protein aggregates are different from disease to disease, this common characteristic shows that protein deposition *per se*, or some related event, is toxic to neurons (Dauer and Przedborski, 2003).

The pathological changes of PD are characterized by degeneration of DA neurons in the substantia nigra and formation of Lewy body (LB) in neurons (Scherfler et al., 2007). Many proteins, including α-synuclein (α-syn), ubiquitin and its related enzymes, aregathered in LB. The study showed that the death of nerve cells in the brain was caused by the α-synprotein conformational change, the formation of amyloid filaments and abnormal accumulation (Dekundy et al., 2015). α-syn is the main component of LB, which is the firstly identified as a protein with gene mutation associated with PD. The abnormal aggregation of the protein is closely related to the pathogenesis of PD (Lubbe and Morris, 2014; Zhang X. et al., 2016). The ubiquitin proteasome system (UPS), a new protein degradation pathway, is regarded as the major pathway of non-lysosomal protein degradation in eukaryotic cells. The study confirmed that the activity of the proteasome dropped substantially in substantia nigra of patients with PD, which weakened the effect of the substantia nigra on the degradation of α-syn and other proteins (Masliah et al., 2005). The overexpression and mutation of α-syn can accelerate mitochondrial disorder, enhance the sensitivity to oxidative stress and promote cell death due to its cytotoxicity mediated by DAT (Alberio et al., 2012).

In a study, rotenone was used to stimulate PC12 cells to develop a cell model of PD with over expression of α-syn. After treatment with bilobalide, the oligomer of α-syn were effectively restrained, cell activity was intensified and apoptosis was decreased accordingly. Before that, some scholars found that the bilobalide could regulate the metabolism of the amyloid precursor protein (APP), increase the proportion of soluble APP alpha, reduce the formation of β-amyloid protein (Shi et al., 2011). The similar experimental results were presented that bilobalide could inhibit the formation of abnormal aggregation of different protein by some common mechanism and alleviate the toxic effects of abnormal proteins on cells. Therefore, it acts as a neuroprotective role in this kind of “protein folding diseases” (Zeng et al., 2013).

In another open study, to produce the symptoms of PD, researchers injected the trace amounts of the proteasome inhibitor lactacystin into the substantia nigra pars compacta (SNC) and ventral tegmental area (VTA) in the brain of SD rats. Compared with the model control, the aggregation of α-syn and the apoptosis of substantia nigra were obviously inhibited in rats with PD after treatment with the Anchanling (Chinese patent medicine), which presented that the mechanism of Anchanling might be related to the improvement of UPS function. When the damaged UPS function was improved, the UPS raised up the degradation of α-syn, thereby reducing the accumulation of intracellular proteins and the formation of inclusion bodies. So, the improving the function of UPS may be of great significance for the prevention and treatment of PD (Gao, 2007; Wu et al., 2009). Some studies have proved that baicalein can protect nerve cells by inhibiting fibrosis procedure of α-syn protein. 12.5 mol·L^−1^ baicalein can significantly inhibit the oligomerization of α-syn and its cytotoxic effect on SH-SY5Y cells (Lu et al., 2011). Clinically, Bushen Huoxue Granule was proved to be effective in treating PD for many years, a Chinese herbal compound granule (Li et al., 2012). The therapeutic mechanisms of Bushen Huoxue Granule against PD might be related with up-regulation of the TrkB expression that could strengthen the effect of repairing nerve injury factors and down-regulation of the Tau expression that could contribute to reduce the condensed expression of proteins in the cells (Yu et al., 2016).

## Conclusion

In summary, PD is regarded as a complex disease caused by interaction among multiple factors (environmental factors, genetic factors) and various mechanisms. Considering curative effect and symptom control, in short term, western medicine is superior to TCM. However, the long-term effect of treatment is debilitated and a series of side effects will be produced. In contrast, TCM has become a research hotspot in recent years due to its the advantages of multiple components and holistic regulation. In particular, some progress has been made in the study of inhibition of oxidative stress, improvement of mitochondrial energy metabolism, resistance to EAA toxicity and suppression of cell apoptosis. A range of TCM is summarized in Table 2, which exhibits neuroprotective effects on dopamine neurons in substantia nigra or shows beneficial improvements on PD symptoms through one or more biological interventions. Although TCM has the glorious history in the treatment of PD, the experimental studies have only been carried out in recent years, especially for the adoption of the PD model. The PD models are mainly divided into two categories: *in vitro*, which includes PC12 cell, SH-SY5Y cell, SN4741, cell and *in vivo*, which includes mouse, rat, zebrafish, Drosophila DJ-1A and so on. Due to the complexity of TCM and its active ingredients, it is difficult to choose the right model to explore its mechanism comprehensively. And, the existing models of PD can only screen some TCM. Therefore, in order to better reveal the pharmacological effects and mechanisms of TCM against PD, several models are firstly applied simultaneously to compensate for the shortage of a single model, and secondly, it is essential to develop more models that conform to the human disease characteristics.

**Table 2:** Summary of TCM on Mechanisms of Anti-PD.

**TCM or extract of TCM**	**Anti-PD mechanisms**	**Model**	**Inducer**	**References**
Anchanling	The inhibition of abnormal protein aggregation; The inhibition of neuronal apoptosis	SD rats	Lactacystin	Gao, 2007; Wu et al., 2009
Baicalein	The inhibition of oxidative stress; The regulation of mitochondrial dysfunction; The reduction of toxic EAA; The inhibition of abnormal protein aggregation	PC12 cells/C57BL/6 mice/SD rats	H_2_O_2_/Rotenone/Rotenone	Zhang et al., 2010, 2017; Lu et al., 2011; Hu et al., 2016
Baichanting Compound	The inhibition of oxidative stress	C57BL/6 mice	MPTP	Ren et al., 2015
Bilobalide	The inhibition of abnormal protein aggregation	PC12 cells	Rotenone	Shi et al., 2011; Zeng et al., 2013
Bushen Huoxue Granule	The inhibition of abnormal protein aggregation	SD rats	6-OHDA	Li et al., 2012; Yu et al., 2016
Carnosic acid	The inhibition of oxidative stress; The inhibition of neuronal apoptosis	SH-SY5Y cells/Wistar rats	6-OHDA/6-OHDA	Wu et al., 2015
Catalpol	The regulation of mitochondrial dysfunction	Kunming mice	Rotenone	Shi et al., 2012
Celastrol	The inhibition of neuroinflammation	Drosophila DJ-1A	–	Faust et al., 2009; Zhang et al., 2012
Curcumin	The inhibition of oxidative stress; The inhibition of neuroinflammation	C57BL/6 mice/Lewis rats	MPTP/Rotenone	Pan et al., 2007; Cui et al., 2016
Forsythia suspense extract	The inhibition of oxidative stress; The inhibition of neuroinflammation	PC12 cell/SD rats	Rotenone/Rotenone	Zhang S. et al., 2016
*Ganoderma lucidium* spore	The inhibition of oxidative stress; The inhibition of neuroinflammation	Wistar rats	6-OHDA	Bao, 2014
Geniposide	The inhibition of neuronal apoptosis	C57BL/6 mice	MPTP	Chen et al., 2015a,c; Zhao et al., 2016
*Ginkgo biloba* Pingchan Recipe	The inhibition of neuronal apoptosis	SH-SY5Y cells/C57BL mice	MPP^+^/MPTP	Wu et al., 2016
Ginsenoside Rg1	The inhibition of neuroinflammation, The inhibition of abnormal protein aggregation	C57BL/6 mice	MPTP&probenecid	Heng et al., 2016
Green tea polyphenols	The inhibition of oxidative stress	C57BL/6J mice	MPTP	Chen et al., 2013
Gui Ling Pa An Wan	The inhibition of oxidative stress; The inhibition of neuronal apoptosis	SD rats	6-OHDA	Meng et al., 2014a,b; Zhang H. Z. et al., 2016
Icariin	The inhibition of neuronal apoptosis	C57BL/6 mice	MPTP	Chen et al., 2017
Isoliquiritigenin	The inhibition of neuronal apoptosis	SN4741 cells	6-OHDA	Hwang and Chun, 2012; Qin and Wu, 2016
Kukoamine A	The inhibition of oxidative stress; The inhibition of neuroinflammation; The reduction of toxic EAA; The inhibition of neuronal apoptosis	SH-SY5Y cells/C57BL/6 mice	MPP^+^/MPTP	Hu et al., 2017
Lycopene	The inhibition of oxidative stress	C57BL/6 mice	Rotenone	Liu et al., 2013
Matrine	The inhibition of oxidative stress	C57BL mice	MPTP	Meng et al., 2017
Paeoniflorin	The inhibition of neuronal apoptosis	C57BL/6 mice	MPTP	Zheng et al., 2017
Paeonolum	The inhibition of oxidative stress; The inhibition of neuronal apoptosis	PC12 cells/zebrafish	MPP^+^	Lu et al., 2015
Parthenolide	The inhibition of neuroinflammation	C57BL/6 mice	MPTP	Zhang H. et al., 2015
Petroleum Ether Extract of *Ficus religiosa* (L.) Leaves	The inhibition of oxidative stress	Wistar rats	6-OHDA	Bhangale and Acharya, 2016
pine bark extract	The inhibition of oxidative stress	C57BL/6 mice	Rotenone	Lu et al., 2014
Piperine	The inhibition of oxidative stress; The inhibition of neuroinflammation; The inhibition of neuronal apoptosis	C57BL/6 mice	MPTP	Yang et al., 2015
Polygona-Polysaccharose	The inhibition of neuroinflammation	SD rats	6-OHDA	Chen et al., 2010
Polyphenols from toona sinensis seeds	The inhibition of neuroinflammation	SD rats	6-OHDA	Li X. J. et al., 2016
Polysaccharide from Spirulina platensis	The inhibition of oxidative stress	C57BL/6J mice	MPTP	Zhang F. et al., 2015
Powder of *Gastrodia elata*	The inhibition of oxidative stress; The inhibition of neuroinflammation	Wistar rats	6-OHDA	Chen et al., 2014; Wang et al., 2014
Proantho cyanidins	The inhibition of oxidative stress	C57BL/6 mice	MPTP	Liang and Zhang, 2016
Protocatechuic acid	The inhibition of oxidative stress	SD rats	6-OHDA	Liu et al., 2013
Protocatechuic acid and chrysin	The inhibition of oxidative stress; The inhibition of neuroinflammation	PC12 cells/zebrafish/mice	6-OHDA/6-OHDA/MPTP	Zhang Z. J. et al., 2015
Puerarin	The reduction of toxic EAA; The inhibition of neuronal apoptosis	C57BL/6 mice	MPTP	Zhu et al., 2012; Huang and Wang, 2015; Jiang et al., 2016
Qianzheng San Extract	The regulation of mitochondrial dysfunction	Kunming mice/C57BL/6 mice	Arecoline Hydrobromide/Oxotremorine/MPTP	Li et al., 2015
Salidroside	The inhibition of oxidative stress; The inhibition of neuronal apoptosis; The inhibition of abnormal protein aggregation	PC12 cells/C57BL/6 mice	MPP^+^/MPTP	Wang et al., 2015
Salvianolic Acid B	The inhibition of oxidative stress; The inhibition of neuroinflammation	Mesencephalic cells/C57BL/6 mice	MPP^+^/LPS/MPTP	Zhou et al., 2014
Schisantherin A	The inhibition of neuronal apoptosis	SH-SY5Y cells/C57BL/6 mice	MPP^+^/MPTP	Sa et al., 2015
Tetrandrine(Tet)	The reduction of toxic EAA	SD rats	6-OHDA	Jin and Bao, 2010
Total flavonoids from *Scutellaria baicalensis*	The inhibition of oxidative stress	C57BL/6J mice	MPTP	Li X. L. et al., 2016
Total Flavonoids of *Clerodendranthus spicatus*	The inhibition of oxidative stress	SH-SY5Y cells/Wistar rats	6-OHDA/6-OHDA	You et al., 2015
Trehalose	The inhibition of abnormal protein aggregation	SD Rats	AAV1/2 A53T α-synuclein	He et al., 2015
Triptolide	The inhibition of neuroinflammation	SD rats	MPP^+^	Hirsch et al., 2005; Gao et al., 2008
Zhenganxifeng decoction	The inhibition of oxidative stress	Wistar rats	6-OHDA	Li X. M. et al., 2016

PD is a result of the interaction of many neuroendocrine factors in the aging state. The use of TCM alone can effectively control the early signs of PD, avoid toxic side effects of western medicine and enhance the compliance of patients with medication greatly. Although TCM have showed the magic effect for the disease, it is difficult to ignore the problem that the composition of the TCM is complex and the mechanism of action is not completely clear. The following suggestions should be particularly considered: (1) more active components should be isolated and screened from TCM, just like artemisinin for malaria; (2) as TCM compound, therapeutic material basis will continue to be searched for the fight against PD, just like compound Danshen dripping pills for coronary heart disease. The compound of active ingredients of TCM, whose material base is relatively clear, adheres to the concept and advantages of formula compatibility of TCM. Thus, it is one of the most important approaches to modern TCM research. With the rapid development of molecular biology, neurobiology, behavioral science and other disciplines of knowledge, single herbs, effective component and TCM compound, through a variety of mechanisms, will create comprehensive and effective prevention and treatment of PD with the anticipation of different time, links, and levels.

## Author contributions

XL summarized the literature and wrote the manuscript. YZ helped with the manuscript writing. YW revised the manuscript. JX wrote part of the manuscript. PX designed the diagram. YM and QW revised the manuscript and provided critical comments. HK supervised all the works. All the authors approved its final version, and agreed to be accountable for all aspects of the work.

### Conflict of interest statement

The authors declare that the research was conducted in the absence of any commercial or financial relationships that could be construed as a potential conflict of interest.

## Abbreviations

TCM: Traditional Chinese medicine
PD: Parkinson's disease
EAA: excitatory amino acids
MPTP: 1-methyl-4-henyl-1,2,3,6-tetrahydropyridine
GSH: Glutathione
MDA: Malondialdehyde
6-OHDA: 6-Hydroxydopamine
PPAR-γ: Peroxisome proliferator-activated receptor-γ
Glu: glutamate
Tet: Tetrandrine
Asp: aspartate
NO: nitric oxide
DA: dopamine
Ach: acetylcholine
GABA: gamma-aminobutyric acid
COX-2: cyclooxygenase-2
LB: Lewy body
α-syn: α-synuclein
UPS: ubiquitin proteasome system.
